# Surgical Reduction and Direct Repair Using Pedicle Screw-Rod-Hook Fixation in Adult Patients with Low-Grade Isthmic Spondylolisthesis

**DOI:** 10.1155/2022/8410519

**Published:** 2022-08-10

**Authors:** Yongjian Gao, Chen Zhao, Lei Luo, Liehua Liu, Lichuan Liang, Dianming Jiang, Pei Li, Qiang Zhou

**Affiliations:** Department of Orthopedic Surgery, The Third Affiliated Hospital of Chongqing Medical University, Chongqing, China

## Abstract

**Background:**

Although direct pars repair using a pedicle screw-rod-hook system has achieved satisfactory results in patients with spondylolysis, its application in adults with low-grade isthmic spondylolisthesis is rarely reported.

**Objective:**

To assess the surgical effect of reduction and direct repair surgery with a pedicle screw-rod-hook system combined with autogenous bone grafts in adult patients with low-grade isthmic spondylolisthesis.

**Methods:**

Sixty-four adult patients with low-grade isthmic spondylolisthesis underwent reduction and direct repair using a pedicle screw-rod-hook system in our department from September 2009 to April 2018. The clinical efficacy was evaluated by clinical and radiological assessments.

**Results:**

The average follow-up was 52.15 ± 9.96 months. The visual analog scale (VAS) scores (VAS-lumbar and VAS-leg) and Oswestry Disability Index (ODI) at the final follow-up (FFU) were significantly lower than the preoperative levels (*P* < 0.05). The modified Prolo score was “excellent” for 60 patients (93.75%) and “good” for 4 patients (6.25%). The slip distance and slipping percentage showed significant decreases postoperatively and FFU compared to preoperatively (*P* < 0.05). There were no significant differences in the disc height, slip angle, and range of motion of the surgical intervertebral space or upper intervertebral space between preoperation and FFU (*P* < 0.05). Successful bony fusion had a 96.86% success rate.

**Conclusion:**

Reduction of slip and direct repair using pedicle screw-rod-hook fixation combined with autogenous iliac bone grafting in adult patients with low-grade isthmic spondylolisthesis is a safe and effective technique.

## 1. Introduction

Spondylolysis is defined as a defect in the pars interarticularis of the lumbar vertebra [1]. The incidence of spondylolysis in the general pediatric and adolescent population ranges from 4.4% to 4.7%, but the prevalence in professional baseball players with back pain is 27.6% [2]. Approximately 39%–82% cases of spondylolysis progress to spondylolisthesis [3, 4]. Isthmic spondylolisthesis (IS) is the anterior translation of one lumbar vertebra relative to the next caudal segment as a result of an abnormality in the pars interarticularis [5]. Adults have an IS incidence of 3.7%–8%, with approximately 60% having low-grade (Meyerding [6] grade I/II) isthmic spondylolisthesis (LGIS) [7]. Symptomatic patients usually present with activity-dependent low back pain (LBP) and/or leg pain with disc degeneration at the slip level. Patients not responding to ≥6 months of conservative treatment or with progressive worsening of symptoms may benefit from surgery [8].

Present, spinal fusion surgery, including interbody fusion and posterolateral fusion, is the most common surgical method in adults with LGIS, with successful arthrodesis and neurologic decompression and established clinical success [9, 10]. However, some patients may undergo reoperation due to adjacent segment disease, pseudoarthrosis, or instrumentation failure [11]. Direct pars repair for LGIS is more reasonable because it restores normal anatomy, preserves spinal segmental motion, and has little effect on the activity of the adjacent segment. There are many types of direct repair, including Buck's operation [12], Nicol and Scott transverse steel wire [13, 14], Morscher hook-screw [15], pedicle screw-rod-hook (PSRH) fixation [16], and other methods, to directly provide internal fixation. The aforementioned methods are mainly used to treat patients with spondylolysis, but rarely for patients with LGIS. Some of the studies have used the PSRH system to treat lumbar spondylolysis, including a small number of patients with slight and low-grade isthmic spondylolisthesis, and obtained satisfactory clinical results [8, 17]. However, few studies have specifically focused on the application of this system to patients with low-grade spondylolisthesis.

The objective of this study was to assess the efficacy of reduction and direct lysis repair by the PSRH system in combination with autogenous bone grafts in 64 patients with LGIS. This is an infrequent retrospective study with a relatively large sample size using the PSRH system to correct olisthesis and repair isthmic lysis in adult patients with LGIS.

## 2. Materials and Methods

### 2.1. Ethics Statement

The hospital ethics committee approved this study and required neither the patient's approval nor informed consent for the review of patients' images and medical records. The data were retrospective in nature and anonymized by the Medical Research Ethics Board. This study was conducted in accordance with the Declaration of Helsinki.

### 2.2. Patient Population

The inclusion criteria were as follows: symptomatic single-segment low-grade (Meyerding grade I/II) bilateral IS; L4 or L5 localization; chronic, disabling LBP possibly radiating to the thighs; no neurological symptoms; discal height of at least two-thirds of its normal height and intervertebral disc degeneration within grade 3 of Pfirrmann's criteria [18]; unresponsive to conservative treatment for at least 6 months; and follow-up of 2 years or more. The exclusion criteria were as follows: IS with lumbar spinal stenosis, lumbar disc herniation, scoliosis, fractures, infection, tumors, severe osteoporosis, and previous lumbar surgery.

Based on the inclusion and exclusion criteria, 64 patients with LGIS were included and underwent surgery from September 2009 to April 2018 in our department (Table 1). All operations were performed by the same surgeon.

### 2.3. Preoperative Management

All patients had anteroposterior lateral, hyperextension, and flexion and both oblique radiographs of the lumbar spine and its computed tomography (CT) scans to confirm the defect of lysis, intervertebral instability, and vertebral slippage. In addition, magnetic resonance imaging (MRI) of the lumbar spine was performed to detect other spinal problems, including disc degeneration and herniation.

### 2.4. Surgical Technique

After a posterior midline longitudinal incision was made, the pars defect, lamina, and starting points of screw insertion were exposed bilaterally by the Wiltse approach [19]. Pars lysis was prepared by removing the fibrocartilaginous defect, and the bony ends and bone cortex of the local pars defect were curetted completely. The autogenous granulated cancellous bone was curetted from a small window of the iliac crest, usually via the same skin incision. Two monoaxial pedicle screws of appropriate length were inserted into the lytic vertebra using a modified technique in which the starting point for the insertion of the pedicle screw was slightly more lateral than usual. Lamina hooks were placed on laminae, and prebent rods according to the curvature of the lamina were placed on the surface of the lamina, connected with the lamina hook and locked. The rod was used as a lever, the facet joint was used as a fulcrum, and the process of tightening screws and rods simultaneously obtained pullout of the slipped vertebral body. Subsequently, the laminar hook and rod were loosened on one side, and the laminar hook and rod were pressurized on the other side to create a compressive force across the pars interarticularis and then locked tightly. Similarly, the hook and rod were compressed to close the isthmic defect on the other side again. Finally, the autogenous granulated cancellous bone was placed on the lysis and fit to the bilateral lamina surface.

### 2.5. Postoperative Care

The patients were allowed to walk with a brace 2 days postoperatively and perform proper functional exercise. After 3 months, patients slowly returned to exercise activities, with a full return to activities at 6 months postoperatively. All patients were examined clinically and radiologically 2-3 days, 3, 6, 12, 18, and 24 months after surgery, and then once a year. When the lumbar spine X-ray showed isthmic union, further lumbar spine CT was performed to confirm the bony fusion of the isthmus. MR was performed at 2 and 4 years after surgery.

### 2.6. Evaluating Standards

#### 2.6.1. Clinical Outcome Assessments

The clinical outcomes were measured using the visual analog scale (VAS) of LBP and lower extremity pain, the Oswestry Disability Index (ODI) of the functional outcome, and the modified Prolo score of the functional and economic statutes [20]. The length of the operation time, the amount of blood loss, and surgical complications were assessed.

#### 2.6.2. Radiological Assessments

The radiological outcomes [21] included the disc height (DH), slip distance (SD), slipping percentage (SP), slip angle (SA), upper intervertebral space angle (UISA), lumbar lordosis (LL), the range of motion of the surgical intervertebral space (ROMSIS), and the range of motion of the upper intervertebral space (ROMUIS).

The above radiographic measurements were measured from the sagittal radiograms and flexion and extension radiographs of the lumbar spine. CT scans were evaluated for bony fusion [22], and MRI scans were evaluated for progressive degenerative changes in the intervertebral disc by Pfirrmann's criteria [18].

### 2.7. Statistical Analysis

All statistical analyses were performed with SPSS version 26.0 statistical software (SPSS, Inc., Chicago, IL, USA). The data conforming to a normal distribution are expressed as the mean ± standard deviation and not conforming as the median ± interquartile range. Clinical outcome assessments and radiological assessments were performed using repeated analysis of variance (ANOVA). The Pfirrmann grading of intervertebral discs was analyzed by the chi-square test. Values of *P* < 0.05 were considered statistically significant.

## 3. Results

### 3.1. Clinical Assessments

General data are given in Table 1, and VAS and ODI values are given in Table 2. The modified Prolo score, representing the ability to restart work and leisure activities, was “excellent” in 60 patients (93.75%) and “good” in 4 patients (6.25%). None of the patients demonstrated a poor outcome.

### 3.2. Radiological Assessments

**Figure 1:**
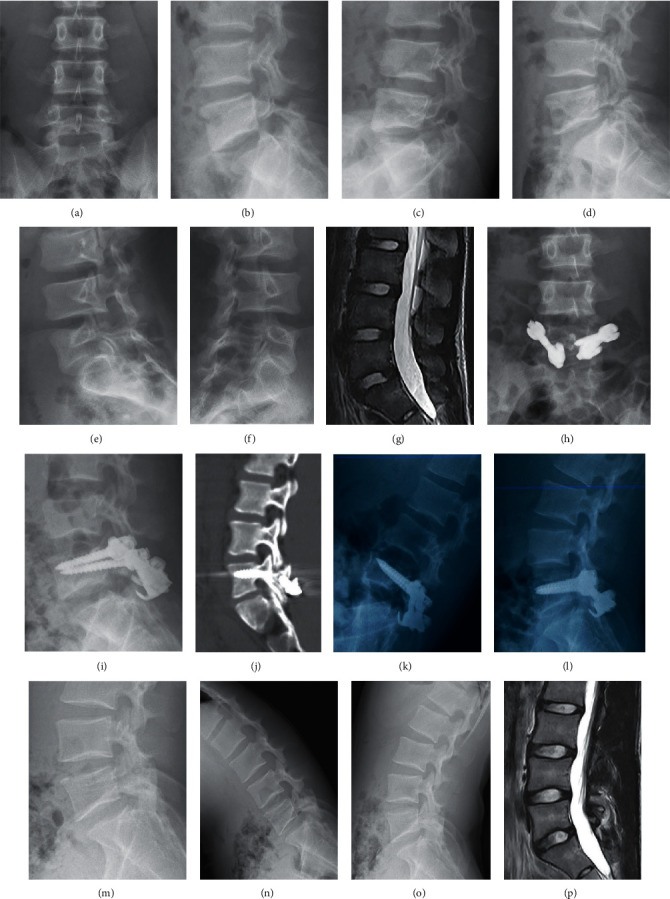
A 16-year-old male patient with LGIS of L5 had LBP of 7 points (VAS scores), without sciatica pain, and underwent direct pars repair using the PSRH system. (a)–(f) The X-rays of the lumbar spine in anteroposterior, sagittal, hyperextension and flexion, and double oblique positions, suggesting LGIS of the L5 with isthmic defect. The disc height and ROM of L5-S1 are 12.95 mm and 23.9°, respectively. (g) The surgical segment and the upper segment are both grade 2 of Pfirrmann's criteria by magnetic resonance imaging (T2-weighted MRI). (h)-(i) The postoperative X-rays, indicating that the isthmic defect has been repaired and lumbar 5 spondylolisthesis has been reset. (j) CT scan of the lumbar spine 12 months after surgery, showing that the isthmic defect has bony fusion. (k)-(l) X-ray films of hyperextension and flexion before removal of internal fixation at 42 months after the operation. (m)–(o) Re-examinations of the lumbar spine X-ray at 78 months after the operation, suggesting that the internal fixation has been removed. The disc height and ROM of the affected level were 10.6 mm and 16.6°, respectively, and the ROM of the upper segment was 12.10°. (p) The lumbar spine MR at the FFU was grade 1 for the L4-5 discs and grades 2-3 for L5-S1 discs.

Radiographic findings are given in Table 3. Successful bony fusion was achieved in 62 cases with 96.86% success rate, and the bony fusion time was 12.00 ± 5.00 months. The disc degeneration assessments by the Pfirrmann grading system are given in Table 4. At the final follow-up (FFU), the Pfirrmann grade of the disc was elevated in 40.63% (26/64) of patients, unchanged in 59.38% (38/64) of patients in the surgical segment, elevated in 25% (16/64) of patients, unchanged in 73.44% (47/64), and decreased in 1.56% (1/64) in the adjacent segment. The FFU of the surgical segment showed significant degeneration of the intervertebral disc preoperatively (*P* ≤ 0.001 < 0.05), while the FFU of the upper segment did not change significantly preoperatively or by FFU (*P*=0.054 > 0.05). A typical case is shown in Figure 1.

### 3.3. Complications

Postoperative complications occurred in 4 patients (6.25%) (Table 5). No implant failure or donor site complications were found in any patient. Nonunion was observed in two cases. One patient underwent reoperation, and another patient was still under follow-up observation. Patients with superficial wound infection or postoperative sciatica recovered after treatment.

## 4. Discussion

According to the recent research, LBP is a major public health problem worldwide, and it remained high between 1990 and 2019 [23]. IS is a common cause of LBP in adolescents and adults [5, 10] and is mainly due to the progression of lumbar bilateral spondylolysis. In adults, most IS present as low-grade isthmic spondylolisthesis (LGIS) (Meyerding I/II) [7]. Patients with persistent symptoms after nonsurgical or interventional injection therapy and patients with severe or progressive neurological dysfunction require surgical treatment.

Direct repair includes Buck's isthmic screwing, Scott's wiring technique, Morscher's hook-screw, and the PSRH system. Direct stereotactic screwing can be challenging for the reduction of slip due to the difficulty in achieving accurate placement of the screws. The Scott technique and hook-screw construct cannot provide strong power to reposition slippage. The Scott technique was used in a small number of cases of low-grade slippage, but the results were not satisfactory [17, 24]. The PSRH system is biomechanically excellent in terms of intervertebral flexion and extension stiffness or intervertebral torsional stiffness in several current surgical methods [25, 26]. Pedicle screws offered stabilization in three columns and high pullout resistance. Therefore, the PSRH system has a strong biomechanical basis for the reduction of the olisthetic vertebral body. A few surgeons have tried to use the PSRH system to treat lumbar spondylolysis cases, including a small number of patients with LGIS, and have achieved satisfactory results [8, 17]. Therefore, we used this method to treat adult LGIS and achieved satisfactory clinical results, including reduction of the slip, bony fusion, and relief from clinical symptoms.

IS combined with isthmic pseudarthrosis is difficult to cure. Spinal fusion surgery focuses on decompression rather than reduction of vertebral translation, and the addition of reduction does not result in better improvements in pain and functions in LGIS [9]. However, reduction is particularly important for the direct repair technique. Reduction of the slippage by the PSRH system reduces the defected area of the isthmus, and the devices provide strong compression force along the axial direction of the lamina, enabling immediate stabilization of the surgical segment after surgery, which is conducive to bony fusion. In this research, the slip distance (SD) and slipping percentage (SP) of the lytic vertebra showed significant decreases postoperatively (SD: 3.07, SP: 7.71%) and FFU (SD: 3.20, SP: 8.01%) compared to preoperatively (SD: 8.78, SP: 21.86%), which showed a satisfactory reduction and maintained it well. The bony fusion rate of the pars was 96.86%, which was even better than the outcomes of spondylolysis research (52%–91%) [22, 27, 28]. The application of the PSRH system achieves local stability of the operative segment, which is good for satisfactory pain relief and finally achieves a satisfactory recovery of lumbar spine function. All patients observed good results regarding pain relief (V.A.S. score) and the clinical outcome (ODI and modified Prolo scores) and a low complication rate. This is consistent with other studies [17].

The imperfection of lumbar fusion surgery sacrifices the mobility of the involved motion, increases the mechanical stress in adjacent segments and subsequent adjacent segment degeneration (ASD), and potentially causes spinal stiffness. In our study, there were no significant differences in the DH, SA, and ROMSIS of the involved segment or LL between the preoperative and FFU values. At the FFU, there was more significant degeneration of the surgical segment than performed preoperatively. A satisfactory result was achieved, showing that direct repair preserved the disc height and mobility of the surgical segment and had little impact on the curvature of the lumbar spine. In patients with significant degeneration of the intervertebral disc before surgery (especially grade 3), the possibility of further degeneration after surgery is likely to be aggravated. DR only repaired the isthmic defect and reconstructed local stability but did not repair the preoperative intervertebral disc degeneration. Only a small part of the stress of the postoperative degenerated intervertebral disc was unloaded, and the disc still bears most of the weight of the body vertically loading the spinal column, and the postoperative intervertebral disc may continue to degenerate. This phenomenon may be the process of natural disc degeneration. At the upper segment, neither UISA nor ROMUIS was significantly different between preoperative and FFU, as well as intervertebral disc degeneration. This suggests that DR does not increase the movement of the adjacent segment and has little effect on adjacent segment degeneration.

In our research, satisfactory reduction of the slip and a high bony fusion rate are highlights of this surgery. Our average operation time was similar to that in the other studies (174.9–198 minutes), but the average blood loss was less than that in those studies (152–468.8 ml) [29, 30]. These advantages are related to the improvement of our surgical approach. First, DR with the PSRH system not only reduced grade I but also part of grade II IS, retained the activity of the operative segment, and had little impact on adjacent segments. Second, to obtain a larger bone graft area and reduce the pedicle tail occupying effect, the pedicle screw placement point of this operation was more lateral than that of the traditional screw placement method and the Wiltse approach could directly reach this site. In addition, the Wiltse approach could reach the surgical site through the natural muscle space, reducing surgical trauma and bleeding. DR using PSRH fixation with satisfactory reduction, adequate bone grafting, less bleeding, and firm internal fixation could ensure a high surgical fusion rate and satisfactory clinical results.

There are a number of limitations to DR with the PSRH system. The method is not suitable for patients who require laminectomy for decompression, such as lumbar disc herniation and lumbar spinal stenosis; with lamina hypoplasia, such as spina bifida; and with high-grade isthmic spondylolisthesis. This system in the reduction of slippage is weaker than the pedicle screw titanium rod system in spinal fusion surgery. Due to the inability to obtain a satisfactory reduction of a high degree of spondylolisthesis, the isthmic defect area is relatively large, and the risk of postoperative pseudoarthrosis or internal fixation failure is high. This study was a relatively small, single-centered, retrospective study. Furthermore, a larger sample size, multicenter, prospective long-term follow-up and the establishment of comparative studies of similar technologies or fusion technologies would be needed.

## 5. Conclusion

Spondylolisthesis reduction and direct repair using pedicle screw-rod-hook fixation combined with autogenous iliac bone grafting in adult patients with low-grade isthmic spondylolisthesis is a safe and effective technique.

## Figures and Tables

**Table 1:** General data (mean ± SD/M ± IQR).

	Direct repair (*n* = 64)/mean ± SD
Age in years (range)	36.50 ± 21.00 (15–51)

Gender, *n* (%)	
Male	36 (56.25)
Female	28 (43.75)

Follow-up in months (range)	52.15 ± 9.96 (32–76)

Leg pain, *n* (%)	14 (21.88)

Level treated, *n* (%)	
L5	51 (79.69)
L4	13 (20.31)

Operation time (range) (min)	187.00 ± 71.00 (120–347)

Blood loss (range) (ml)	65.00 ± 31.00 (30–180)

*n*, number of patients; SD, standard deviation; M, median; IQR, interquartile range. *Note.* Values in the age, operation time, and blood loss data represent M ± IQR, and follow-up time represents mean ± SD.

**Table 2:** Clinical outcome assessments (M ± IQR).

	Preoperative	Postoperative	FFU
VAS-LBP	6.00 ± 1.00^ab^	2.00 ± 1.00^ac^	0 ± 1.00^bc^
VAS-leg	3.00 ± 1.25^ab^	0 ± 1.00^a^	0 ± 0^b^
ODI	48.00 ± 6.25^ab^	20.00 ± 7.30^ac^	10.00 ± 8.00^bc^

ODI, Oswestry Disability Index; VAS, visual analog scale (VAS); LBP, low back pain; FFU, final follow-up. ^a^Preoperative vs. postoperative (*P* < 0.05); ^b^preoperative vs. FFU (*P* < 0.05); ^c^postoperative vs. FFU (*P* < 0.05). *Note.* Values in the VAS-LBP, VAS-leg, and ODI data represent M ± IQR.

**Table 3:** Radiological assessments (mean ± SD/M ± IQR).

	Preoperative	Postoperative	Postoperative 6 months	FFU
DH (mm)	12.33 ± 2.25^*∗*^	14.12 ± 2.16^*∗*^^¢£^	12.90 ± 2.11^¢¤^	12.04 ± 2.12^£¤^
SD (mm)	8.80 ± 3.50^*∗*^^#&^	3.50 ± 5.10^*∗*^	3.70 ± 5.00^#^	3.80 ± 5.00^&^
SP (%)	21.88 ± 7.90^*∗*^^#&^	8.70 ± 13.00^*∗*^	9.13 ± 12.60^#^	9.08 ± 12.20^&^
SA (°)	12.16 ± 6.24^#^	11.23 ± 5.42	10.28 ± 5.09^#^	10.58 ± 4.80
UISA (°)	12.07 ± 2.58^*∗*^	9.31 ± 2.78^*∗*^^¢£^	11.00 ± 2.95^¢^	11.39 ± 2.56^£^
LL (°)	52.16 ± 12.39^*∗*^^#^	41.82 ± 12.72^*∗*^^¢£^	48.90 ± 16.72^#¢¤^	54.35 ± 14.20^£¤^
ROMSIS (°)	13.62 ± 6.65^#^	—	7.63 ± 4.80^#¤^	11.50 ± 9.50^¤^
ROMUIS (°)	10.46 ± 5.45	—	8.74 ± 4.39^¤^	10.81 ± 4.96^¤^

DH, disc height; SD, slip distance; SP, slipping percentage; SA, slip angle; UISA, upper intervertebral space angle; LL, lumbar lordosis; ROMSIS, range of motion of the surgical intervertebral space; ROMUIS, range of motion of the upper intervertebral space. ^*∗*^Preoperative vs. postoperative (*P* < 0.05); ^#^preoperative vs. post-6 months (*P* < 0.05); ^&^preoperative vs. FFU (*P* < 0.05); ^¢^postoperative vs. post-6 months (*P* < 0.05); ^£^postoperative vs. FFU (*P* < 0.05); ^¤^post-6 months vs. FFU (*P* < 0.05). *Note.* Values in SA, UISA, ROMUIS, and DH results except for postoperative 6 months, LL results except for postoperative 6 months and the last follow-up, and ROMSIS results except for the last follow-up represent mean ± SD, and other results use M ± IQR.

**Table 4:** Lumbar disc degeneration observed on MRIs by the Pfirrmann grading system.

Pregrades	FFU of SSD (*n*)					FFU of USD (*n*)				
	1	2	3	4	5	1	2	3	4	5
1	2	3				7	1			
2		16	2				35	13		
3			20	21			1	5	2	

*N*, number of patients; FFU, final follow-up; SSD, surgical segmental disc; USD, upper segmental disc.

**Table 5:** Complications.

Postoperative complications	*n* (%), *N* = 64
Nonunion	2 (3.13%)
Postoperative sciatica	1 (1.56%)
Superficial wound infections	1 (1.56%)

*N* is the total number of patients; *n* is the number of patients with complications.

## Data Availability

The data used to support this study are included within the article.
